# Supplementary material for: The interactome of intact mitochondria by cross-linking mass spectrometry provides evidence for coexisting respiratory supercomplexes
Source: Mol Cell Proteomics. 2017 Dec 8;17(2):216–32. doi: 10.1074/mcp.RA117.000470 (PMC5795388; doi:10.1074/mcp.RA117.000470)
Supplement: Supplemental Data [file supp_RA117.000470_133922_0_supp_23978_fzffwf.zip › spectra_annotation/mito_spectra_annotation/359-1-14-1-4-1.pdf]

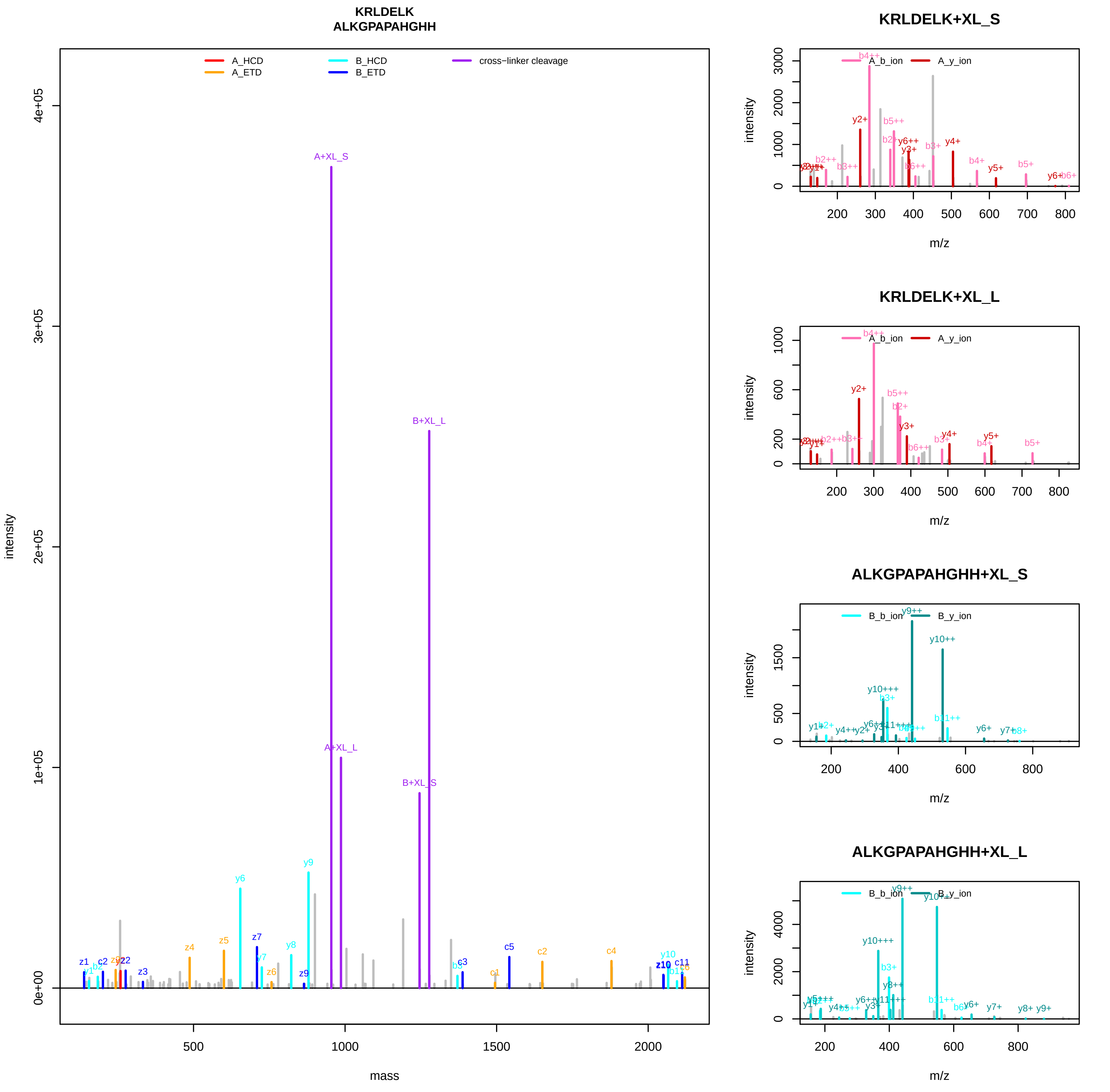
KRLDELK
ALKGPAPAHGHH
A_HCD
A_ETD
B_HCD
B_ETD
cross−linker cleavage
intensity
mass
0e+00
1e+05
2e+05
3e+05
4e+05
500
1000
1500
2000
A+XL_S
B+XL_L
A+XL_L
B+XL_S
y6
y9
y8
y7
z7
z5
z4
z6
z9
z3
z1
c2
c3
c5
c2
c4
c1
b3
y10
z10
c11
KRLDELK+XL_S
KRLDELK+XL_L
ALKGPAPAHGHH+XL_S
ALKGPAPAHGHH+XL_L
A_b_ion
A_y_ion
B_b_ion
B_y_ion
m/z
y9++
y10++
y10+++
b3+
b11++
y6+
y7+
b8+
b4++
y2+
b5++
b2+
y3+
y4+
y5+
b4+
b5+
y6+
b6+
